# Biological effects of exposure to 2650 MHz electromagnetic radiation on the behavior, learning, and memory of mice

**DOI:** 10.1002/brb3.3004

**Published:** 2023-04-28

**Authors:** Rongqi Zheng, Xianxie Zhang, Yan Gao, Dawen Gao, Wenjing Gong, Chenggang Zhang, Guofu Dong, Zhihui Li

**Affiliations:** ^1^ Laboratory of Electromagnetic Biological Effects, Beijing Insititute of Radiation and Medicine Beijing China; ^2^ Department of Pharmaceutical Sciences, Beijing Institute of Radiation and Medicine Beijing China

**Keywords:** anxiety, brain‐derived neurotrophic factor, electromagnetic radiation, gamma‐aminobutyric acid, glucocorticoid, hippocampus, memory

## Abstract

**Background:**

With the development of communication technology, the public is paying increasing attention to whether electromagnetic radiation is harmful to health. Mobile phone communication has entered the 5G era, and there are almost no reports on electromagnetic radiation at 2650 MHz. Therefore, it is necessary to evaluate the risk of adverse effects of 5G mobile phone EMR exposure on the human brain.

**Methods:**

Male animals were continuously exposed to 2650 MHz‐EMR for 28 days with a whole‐body averaged specific absorption rate (WBSAR) of 2.06 W/kg for 4 h per day. Mouse behavior was assessed using the open‐field test (OFT), elevated‐plus maze (EPM), and tail suspension test (TST). The Morris water maze (MWM), HE staining, and TUNEL staining were used to evaluate the spatial memory ability and pathological morphology of hippocampal dentate gyrus cells. Additionally, the expression levels of brain‐derived neurotrophic factor (BDNF), aminobutyric acid (GABA), and glucocorticoid (GR) in the hippocampus were detected by western blotting and immunohistochemistry, while the corticosterone (CORT) level in serum was detected by ELISA.

**Results:**

In the OFT, the total distance traveled, central distance traveled, and residence time significantly decreased in the EMR exposure group (*p* < .05). In EPM, the percentage of the number of times to open the arm and the percentage of time to open the arm significantly decreased in the EMR exposure group. However, in the TST, the two groups had no significant difference in the 4‐min immobility time. In the MWM, the escape latency of the EMR exposure group was shorter than that of the control group, with no significant difference. Furthermore, CORT levels in serum were significantly increased in the EMR exposure group (*p* < .05), while the expression of BDNF and GR proteins in the hippocampus was reduced (*p* < .05), but there was no significant difference in GABA expression.

**Conclusions:**

Our results indicate that exposure to 2650 MHz‐EMR (WBSAR: 2.06 W/kg, 28 days, 4 h per day) had no significant effect on the spatial memory ability of mice (in comparison to little effect). The exposure may be associated with anxiety‐like behavior in mice but not related to depression‐like behavior in mice.

## Graphical abstract

Our results indicate that exposure to 2650 MHz‐EMR (WBSAR: 2.06 W/kg, 28 days, 4 hours per day) has no significant effect on the spatial memory ability of mice (in comparison to little effect). It may be associated with anxiety‐like behavior in mice but not related to depression‐like behavior in mice.

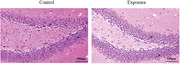

## 1 BACKGROUND

In the process of using mobile phones and other wireless communication devices, people are inevitably exposed to electromagnetic radiation (EMR). EMR refers to the phenomenon in which electromagnetic energy is radiated into space in the form of electromagnetic waves (Rohrlich, 1961). The radiofrequency electromagnetic field (RF‐EMF) is electromagnetic radiation with a frequency range of 100 kHz to 300 GHz (International Commission on Non‐Ionizing Radiation Protection [ICNIRP], 2020). Due to the exponential increase in the use of wireless personal communication devices (e.g., mobile or cordless phones and Wi‐Fi or Bluetooth‐enabled devices) and the infrastructure facilitating them, exposure to radiofrequency electromagnetic radiation levels in the 1 GHz band has increased by approximately 10^18^ times from extremely low natural levels (Bandara & Carpenter, 2018). To fully protect the health of the general public and those who specialize in the electromagnetic environment, the International Commission on Non‐Ionizing Radiation Protection (ICNIRP) has formulated guidelines. In the frequency range of 100 kHz‐6 GHz, the whole‐body averaged specific absorption rate (WBSAR) is 0.08 W/kg, and the SAR value of local limb exposure is 4 W/kg for public exposure, while WBSAR is 0.4 W/kg, and the SAR value of local limb exposure is 20 W/kg for occupational exposure ( International Commission on Non‐Ionizing Radiation Protection [ICNIRP], 2020). With the continuous development of communication technology and improved living standards, mobile phones have become indispensable to modern people. However, to date, research on the electromagnetic radiation of mobile phones has mainly focused on the frequency band between 900 MHz and 1800 MHz, which corresponds to the working frequency band before the fourth generation of mobile communication technology (Fragopoulou et al., 2010; Narayanan et al., 2009; Xu et al., 2006). There are no relevant studies to clarify the health effects of 5G mobile phone radiation.

Evidence from previous studies indicates that exposure to EMR in experimental animals can cause changes in their behaviors, such as EMR (1.8 GHz), which is associated with anxiety‐like and depression‐like behaviors in mice (Gupta et al. 2019; Zhang et al., 2017). Yang et al. (2022) found that there was no significant difference in static or dynamic functional network connectivity in both real and sham exposure conditions and noted that the impact of short‐term electromagnetic exposure was undetected at the ICNs level. These changes depend largely on the duration, frequency, and intensity of EMR exposure. For example, exposure to low‐frequency (50 Hz, 24 weeks) EMR was not associated with obvious anxiety and depression in male rats (Lai et al., 2016; Tang et al., 2015); exposure to high‐frequency (9.417 GHz, 5 weeks) EMR caused anxiety‐like behaviors (Zhang et al., 2015). EMR (900 MHz) can also affect the morphological changes in the amygdala of rodents (Narayanan et al., 2018), which can regulate the hypothalamic‒pituitary‒adrenal (HPA) axis, leading to anxiety‐like behavior (Pawlak et al., 2003). The hippocampus, as the high‐level regulating center of the stress response of the HPA axis, can not only suppress the stress response of the HPA axis but also play a negative feedback regulatory role on the HPA axis. The hippocampus is rich in glucocorticoid receptors (GRs). When stress increases HPA axis function, the level of glucocorticoids will increase, which will cause the GR density in the hippocampus to decrease. Some clinical reports indicate that GR, corticotropin‐releasing hormone‐2 (CRH‐2) and corticosterone are involved in maintaining homeostasis and regulating stress and anxiety (Tinnikov, 1999). Although there are some studies on the relationship between EMR (2450 MHz) and oxidative stress or between EMR and anxiety‐like behaviors (Sinha, 2008), there are relatively few studies on the expression of proteins related to EMR and anxiety‐like behaviors.

There are some studies on mobile phone EMR that have suggested brainwave changes after EMR exposure (Bak et al., 2010; Maganioti et al., 2010). For example, Lustenberger et al. (2015) studied 20 young male subjects who were exposed twice for 30 min prior to sleep to the same amplitude of modulated 900 MHz (2 Hz pulse, 20 Hz Gaussian low‐pass filter and a ratio of peak‐to‐average of 4) RF‐EMF (spatial peak absorption of 2 W/kg averaged over 10 g) 2 weeks apart. The topographical analysis of EEG power during all‐night nonrapid eye movement sleep revealed exposure‐related increases in the delta‐theta frequency range in several frontocentral electrodes and no differences in the spindle frequency range. Yang et al. (2017) showed that exposure to long‐term evolution EMF reduced the spectral power and the interhemispheric coherence in the *alpha* and *beta* bands of the frontal and temporal brain regions. No significant change was observed in the spectral power and the interhemispheric coherence in different timeslots during and after the exposure. Similarly, research has reported that pulsed‐RFR‐exposure‐related (900 MHz, 2 Hz pulse, peak spatial SAR 2 W/kg over 10 g tissue, 30 min) increases in the delta‐theta EEG frequency range in several frontocentral brain areas in humans during non‐REM sleep (Lustenberger et al., 2015). Exposure to EMR can change the permeability of the blood‒brain barrier, which may be related to the death of brain cells (Eberhardt et al., 2008). According to reports, several studies have documented that EMR exposure may be related to changes in the hippocampal structure of mice, and one of the reasons for cognitive impairment in EMR (900 MHz) (Ahmed et al., 2017). The escape latency of the rats in the radiation group was significantly increased, which indicates that the spatial memory ability of the rats was impaired (Narayanan et al., 2015). In contrast, some researchers suggest that EMR (918 MHz) can improve the symptoms of neurodegenerative diseases (Arendash et al., 2010; Dragicevic et al., 2011), such as Alzheimer's disease, which can reduce the deposition of amyloid‐β (Aβ) in the brain and increase neuronal activity and cerebral blood flow (Arendash et al., 2010).

In this study, we used an electromagnetic reverberation chamber (RC) to simulate the 5G mobile phone frequency (2650 MHz, 4 h per day for 28 days, WBSAR: 2.06 W/kg) and explore the biological effects of EMR exposure on behaviors and spatial memory in male mice.

## 2 MATERIALS AND METHODS

### 2.1 Animals

Male C57BL/6 N mice (6–8 weeks, average weight ± standard deviation: 20.26±0.89 g) were obtained from Beijing Weitong Lihua Experimental Animal Technology Co., Ltd. (SCXK [Beijing]‐2015‐0001). During the study, mice were kept in the laboratory environment under 12/12 h day/night periods, 22°C room temperature, and 60% relative humidity. The disposal of animals during the experiment strictly abided by the “Guiding Opinions on Treating Experimental Animals” promulgated by the Experimental Animal Welfare Ethics Committee of National Beijing Drug Safety Evaluation and Research Center and provided humanitarian care according to the 3R principle used by experimental animals.

### 2.2 Electromagnetic radiation exposure system

**FIGURE 1:**
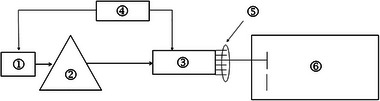
Reverberation chamber radiation exposure system. 1: signal source; 2: radiofrequency power amplifier; 3: antenna selector; 4: control computer; 5: connection cables of the same length; 6: reverberation chamber.

The RC consists of a signal source, radiofrequency power amplifier, wide‐band antenna, antenna selector, metal box, metal stirrer control meter, double‐layer animal stand, animal irradiation container, control machine (computer), control software (application program), and corresponding cable composition (Figure 1). The RC can carry out whole‐body exposure of animals and operates from 0.8 to 4 GHz. The details of the RC can be found in the published literature (Min et al., 2018). Moreover, the shield walls are made of metallic mesh that can be used for ventilation and heat dissipation without the aid of other equipment. In this study, six antennas were connected to different positions of the signal converter (R16‐SN12T18‐D‐BP, China), and the microwave beam was output from one of the antennas to the RC in turn. They could illuminate the RC alternatively and randomly, with an interval of 1 s. The exposure frequency was 2650 MHz, and the targeted dosimetric value was 2 W/kg (WBSAR). There were two kinds of simulation‐measurement methods used to obtain the necessary field (Chakarothai et al., 2013; Gong et al., 2017). In brief, the studies computed WBSAR in small animals using either 12 plane waves with specific configurations or random incident waves. The ratio between the averaged E‐field strength and WBSAR was therefore derived. By measurement, once the realistic E‐field strength in the experiment was recorded, the realistic WBSAR could be obtained. We used an EF0391 electric field probe (Narda, Germany) to measure the field strength (V/m) during actual exposure to ensure that the expected field strength for WBSAR was achieved. In the exposure experiment, a field strength of 78.9 V/m was sufficient to generate WBSAR as 2.06 W/kg with 10 mice weighing 20.0 g.

### 2.3 Experimental design

After 7 days of adaptation to each experiment, the 20 male C57BL/6 N mice were randomly divided into a control group (*n* = 10, average weight 19.96±1.07 g) and an exposure group (*n* = 10, average weight 20.04 ± 0.71 g) according to their body weight. The experiment was repeated three times with a total of 60 male C57BL/6 N mice. The mice in the exposure group were exposed consecutively to RC for 28 days, 4 h (from 8:00 AM to 12:00 AM) each day (Djordjevic et al., 2015; Varghese et al., 2018). In addition to not being exposed to electromagnetic radiation, we placed the control mice in the same radiation cage for 4 h to minimize the impact of restraint stress. Twenty‐four hours after 28 days of EMR exposure, the corresponding behavioral tests, including anxiety‐like behavior assessment using the open‐field test (OFT), elevated‐plus maze (EPM), and depression‐like behavior assessment using the tail suspension test (TST), were assessed. Spatial learning behavior assessment using the Morris water maze (MWM) was performed 48 h after 28 days of EMR exposure.

### 2.4 Open‐field test

**FIGURE 2:**
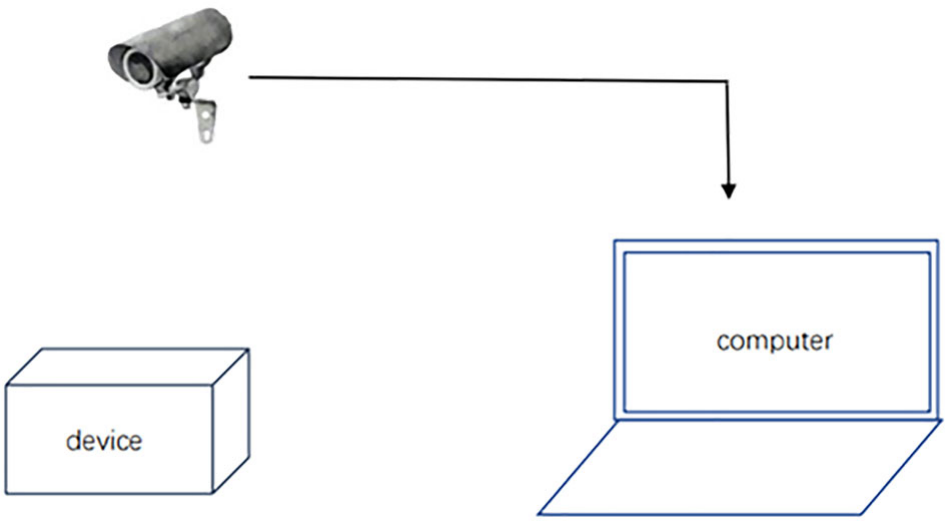
Schematic diagram of the open‐field test.

The open‐field test device consisted of a polypropylene ester open box (length 50 cm, width 50 cm, height 40 cm), as shown in Figure 2. The bottom was divided into 16 grids, and the middle four grids served as the central zone. During the experiment, the mice were placed in a box at a fixed corner to allow them to move freely and then immediately began recording the spontaneous activity of the mice within 5 min. After the end of each test, the feces and urine left by the mice were cleaned and dried with 75% alcohol.

### 2.5 Elevated‐plus maze

**FIGURE 3:**
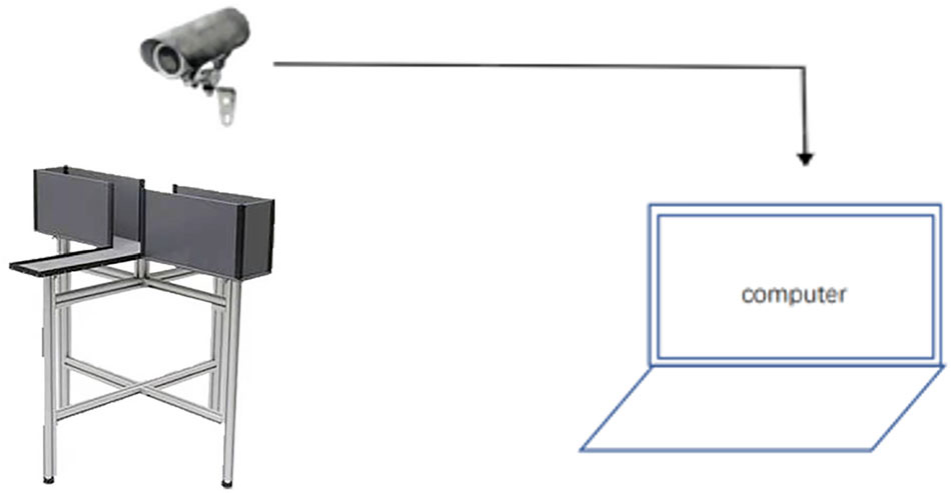
Schematic diagram of the elevated‐plus maze.

The elevated‐plus maze device, which consists of two open arms (length 50 cm, width 5 cm) and two closed arms (length 50 cm, width 5 cm, height 15 cm), is located off the ground, as shown in Figure 3. At the beginning of the experiment, the mice were placed at the junction of the open arms and closed arms with the mouse head facing the side of the open arms. The video was then recorded for 5 min. The number and time of the mice entering the open and closed arms were counted, and the percentage of the number of times the mice entered the open arms was counted as the number of times the mice entered the open arms/the total number of arms entered × 100% and open arms residence time/total arms entrance time x 100%. After the end of each mouse test, the feces and urine left by the mice were cleaned and dried with 75% alcohol.

### 2.6 Morris water maze

**FIGURE 4:**
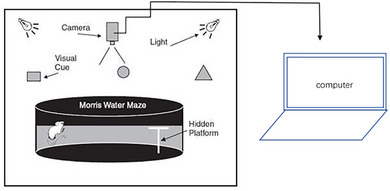
Schematic diagram of the Morris water maze.

The water maze test followed previous reports (Vorhees & Williams, 2006). The water maze was 120 cm in diameter and 50 cm in height, as shown in Figure 4. The water temperature was kept at 21 ± 1°C. A camera was placed above the water to record the movement track of the mouse. The pool was divided into four quadrants. The underwater hidden platform was placed 1.5 cm below the 4th quadrant level. The starting point of the four quadrants was the entry point. When the mouse entered the water, its head faced the pool wall. Reference objects and lighting conditions outside the maze remained unchanged during training. Acquisition phase: the mice were sequentially placed into the pool from the 4 quadrants, and the time to find the platform within 60 s was recorded. If the mouse did not find the platform within 60 s, it was towed to the platform and stayed on the platform for 10 s. The escape latency was recorded as 60 s, and each training interval was 30 s. Acquisition phase training was performed on five consecutive days. The escape latency on the fifth day was used as the final score, and the road map of the mouse to find the platform was observed and recorded (D'hooge & De Deyn, 2001). Probe trial: On day 6, the underwater hidden platform was removed, and a water entry point was randomly selected to record the number of times the mouse crossed the original platform within 60 s and the residence time of the quadrant in the original platform to detect the learning and memory ability (Nunez, 2008).

### 2.7 Tail suspension test

During the experiment, the tail of the mouse was glued at approximately the 1/3 the length with medical adhesive cloth and suspended in the observation box of the animal behavior analysis system 30 cm away from the ground so that the mouse was in an upside down state. The state of the mouse was recorded within 6 min, and the immobile time of the mouse was analyzed within 2–6 min.

### 2.8 Serum corticosterone level test

At the end of all behavioral experiments, trunk blood was collected between 9:00 AM and 11:00 AM in the morning. The blood was left at room temperature for approximately 30 min and centrifuged at 4000 r/min at 4°C for 15 min, and the supernatant was collected. The serum CORT level was detected by an ELISA kit (ml037564, Shanghai MLBIO Biotechnology Co. Ltd).

### 2.9 Histopathological studies

At the end of the behavioral test, five mice were randomly selected from each group of mice. After anesthesia was intraperitoneally injected with 1% pentobarbital sodium (50 mg/kg), 4% paraformaldehyde was perfused through the heart, and the brain was decapitated. After fixation for 24 h, the area of the hippocampus after –4.6 to –2.6 mm was removed from the crown before coronation, followed by a series of dehydrations and paraffin coating, and the slices were 5 μm thick. The sections were subjected to conventional HE staining, and the number and morphological changes of cells in the hippocampus were observed under a light microscope. Cell morphology was quantitatively determined by the depth of hematoxylin staining and the volume of individual cells. Four mouse brains in each group were used for paraffin sectioning, and five white slices were cut from each brain for different experiments.

### 2.10 Western blot analysis

Fresh hippocampal tissues were taken from the two groups of mice, and lysate (containing an appropriate amount of protease inhibitor) was added. After grinding the tissues with a tissue homogenizer, supernatant protein was collected after centrifugation at 4°C and 12,000 r/min for 10 min. The protein concentration was determined by the BCA method, and the total protein content was 40 μg for 12.5% polyacrylamide gel electrophoresis. The protein was transferred to a polyvinylidene difluoride (PVDF) membrane by the wet electric transfer method. After the 5% skim milk powder was blocked for 4 h, primary anti‐BDNF (Boster Biological Technology Co. Ltd, 37 KD), GABA (Abcam, 50 KD), and Tubulin (Boster Biological Technology Co. Ltd, 55 KD) antibodies were added at a ratio of 1:50, 1 μg/mL, and 1:500, respectively, and incubated overnight at 4°C. After PBST washing of the membrane, 2.5% skim milk was incubated with the secondary antibody (goat against mouse [1:5000], goat against rabbit [1:5000])) at room temperature for 1 h. After PBST washing of the membrane, the ECL luminescence method was used for color display, and images were treated by the ImageLab gel imaging system.

### 2.11 Immunohistochemical analysis

Brain tissue specimens were dewaxed with xylene twice and then dehydrated with anhydrous ethanol. After water and PBS rinses as well as 3% hydrogen peroxide treatment, an additional PBS rinse was performed three times after antigen repair. Goat serum working solution was then added to the samples and incubated at 37°C for 30 min. The GR antibody (Boster Biological Technology Co. Ltd. [1:50]) was added to the samples and incubated overnight at 4°C. After washing with PBS, goat anti‐rabbit working solution was added to the specimens and incubated at 37°C for 60 min. DAB staining was performed (2 min), and when the cytoplasm showed a notable brown color under a microscope, staining was stopped. After washing the film thoroughly, it was dehydrated, transparent, and sealed with neutral resin (Biesterfeld et al., 2003). Image‐Pro Plus 6.0 software was used to analyze the integrated optical density (IOD) of GR‐positive expression.

### 2.12 TUNEL staining

The sample was washed twice with xylene and then dehydrated with ethanol, treated with Proteinase K working solution at 4°C for 10 min, and 50 μL TUNEL reaction mixture was added to react at 37°C for 60 min. After PBS rinsing, 50 μL converter‐POD was added and reacted in a dark wet box at 37°C for 20 min, and 50 μL DAB was added and reacted at 37°C for 10 min. Hematoxylin was counterstained, washed with water, dehydrated with ethanol, xylene transparent, and then sealed with neutral resin.

### 2.13 Statistical analysis

We used SPSS 23 for data analysis. If the data satisfied normality and homogeneity of variance, the independent sample *t* test was used; otherwise, the nonparametric test (Mann‒Whitney *U* test) was used. Repeated measures analysis of variance (ANOVA) was performed on data recorded across time periods. The results are expressed as the mean ± SD, and *p* < .05 was considered statistically significant.

## 3 RESULTS

### 3.1 Changes in mouse weight before and after exposure

**FIGURE 5:**
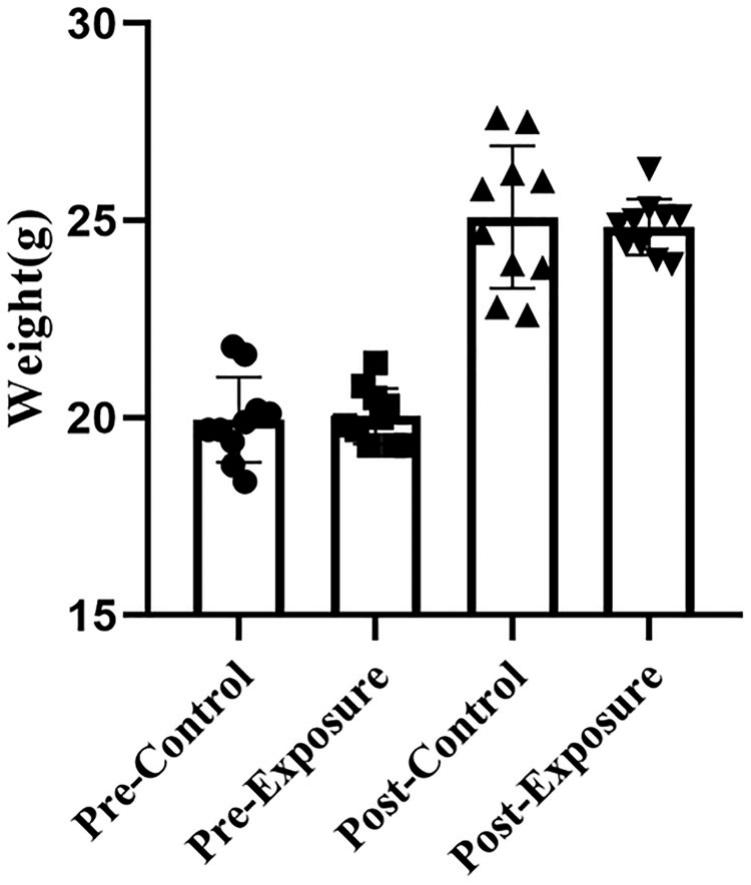
Changes in mouse weight before and after exposure (*n* = 10). All results are expressed as the mean ± SD, and there was no significant difference between the control and exposure groups (*p* > .05).

From Figure 5, we can see that before exposure, the mice's weight of the control group was 20.0 ± 1.07 g, and the exposure group was 20.0 ± 0.71 g. After exposure, the mice's weight of the control group was 25.1 ± 1.81 g, the exposure group was 24.8 ± 0.71 g, and the two showed no difference (*p* = .576).

### 3.2 Effect of 2650 MHz‐EMR on the anxiety‐like behavior of mice

**FIGURE 6:**
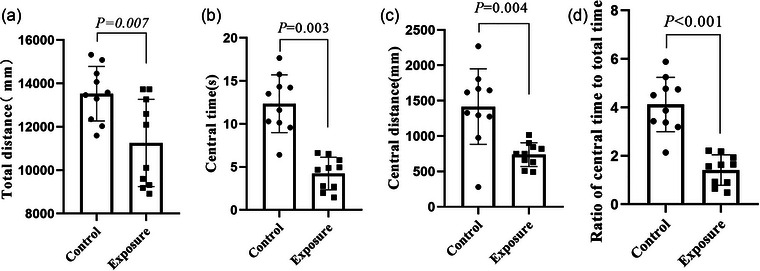
The effect of 2650‐MHz‐EMR exposure on mice (*n* = 10). (A) Total distance; (B) central time; (C) central distance in the open‐field test; (D) ratio of central time to total time. All results are expressed as the mean ± SD, and there was a significant difference between the control group and the exposure group (*p* < .01).

To investigate the effects of exposure to 2650 MHz‐EMR on behavior in mice, we used the open‐field test and elevated‐plus maze to evaluate whether the mice had anxiety‐like behavior. In the OFT, the total distance traveled by the mice in the EMR exposure group was significantly decreased (*p* = .007) compared with the mice in the control group after receiving 28‐day 2650‐MHz‐EMR exposure, indicating that the mice in the exposure group had decreased locomotor activity (Figure 6A). Furthermore, 2650‐MHz‐EMR exposure was associated with a significant decrease in the amount of time spent in the center (*p* = .003), center area distance (*p* = .004), and percent time spent in the center (*p =* .0005) in the OFT compared with the control group (Figure 6B and C). These results suggest that exposure to 2650 MHz‐EMR is associated with anxiety‐like behavior in mice.

**FIGURE 7:**
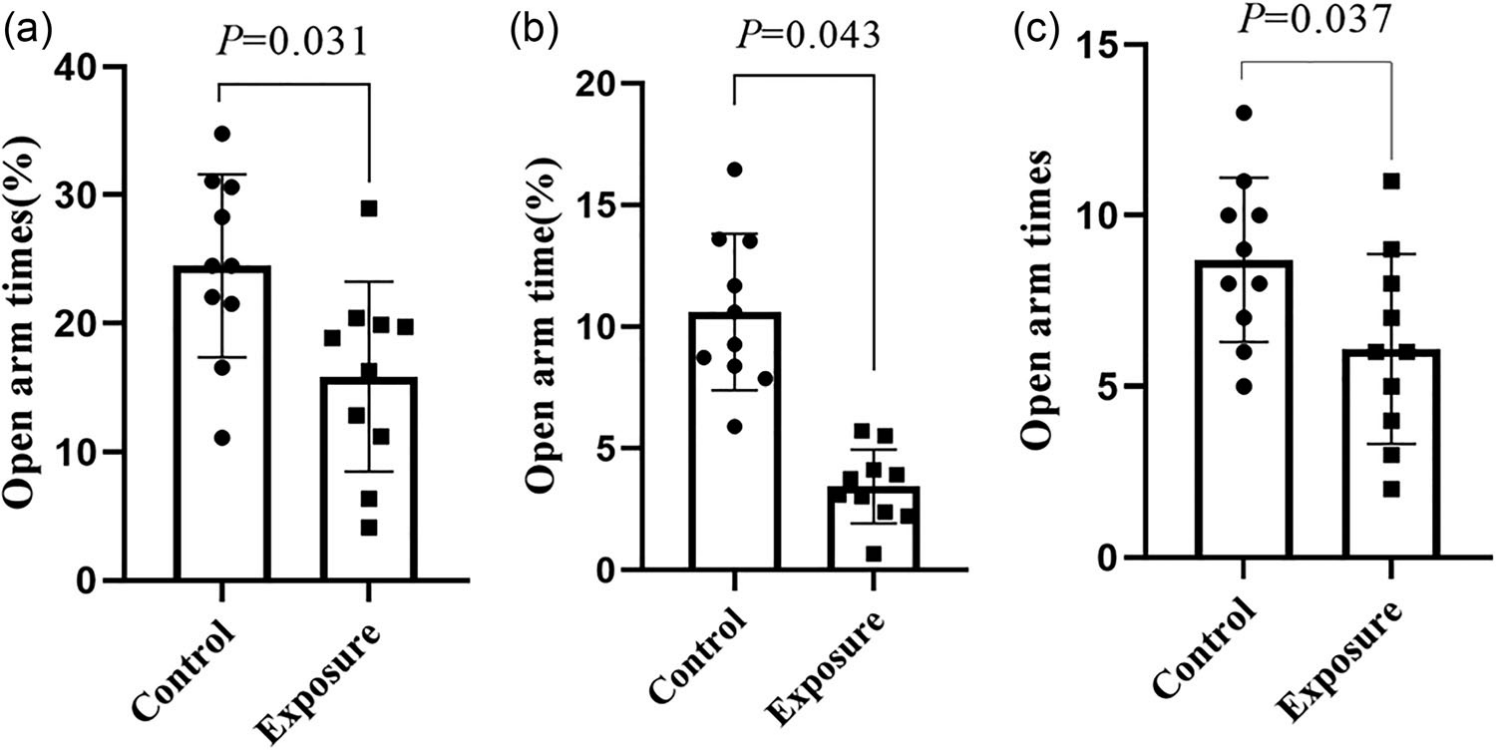
The effect of 2650‐MHz‐EMR exposure on mice (*n* = 10). (A) Percentage of open arm entries; (B) time spent in the open arm in the elevated‐plus maze; (C) the number of times in open arm. All results are expressed as the mean ± SD, and there was a significant difference between the control and exposure groups (*p* < .05).

To further verify the anxiety‐like behaviors induced by 2650‐MHz‐EMR exposure, we carried out the elevated‐plus maze. Our results show that the percentage of the number of times to enter the open arm (*p* = .031), the percentage of open arm retention time (*p* = .043), and the number of times in open arm (*p* = .037) in the EMR exposure group were significantly lower than those in the control group (Figure 7A–C). This is consistent with the results of the OFT. This result suggests that exposure to 2650 MHz‐EMR is associated with anxiety‐like behavior in mice.

**FIGURE 8:**
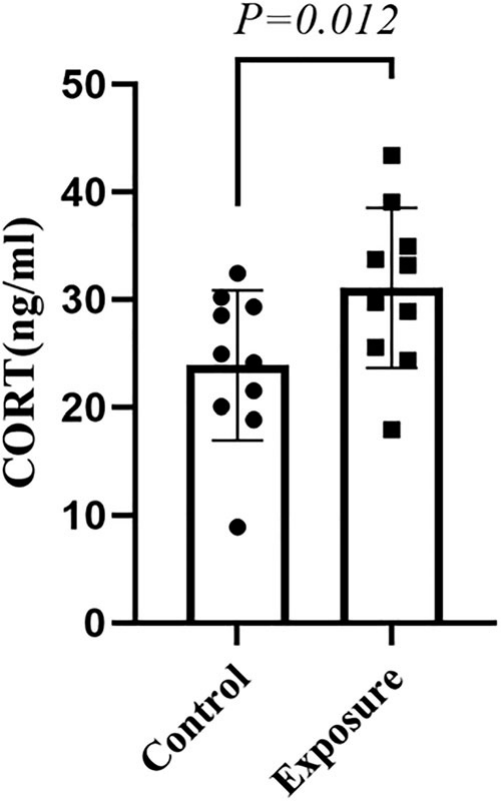
Effects of 2650‐MHz‐EMR exposure on serum corticosterone in mice (*n* = 10). All results are expressed as the mean ± SD, and there was a significant difference between the control and exposure groups (*p* < .05).

Since the OFT and EPM behavior results showed anxiety‐like behaviors in the exposure group, we further verified anxiety‐like behaviors at the molecular level in mice. The results showed that exposure to 2650 MHz‐EMR significantly increased the level of serum corticosterone compared to the control group (*p* = .012), as shown in Figure 8.

### 3.3 Effect of 2650 MHz‐EMR on the spatial memory of mice

**FIGURE 9:**
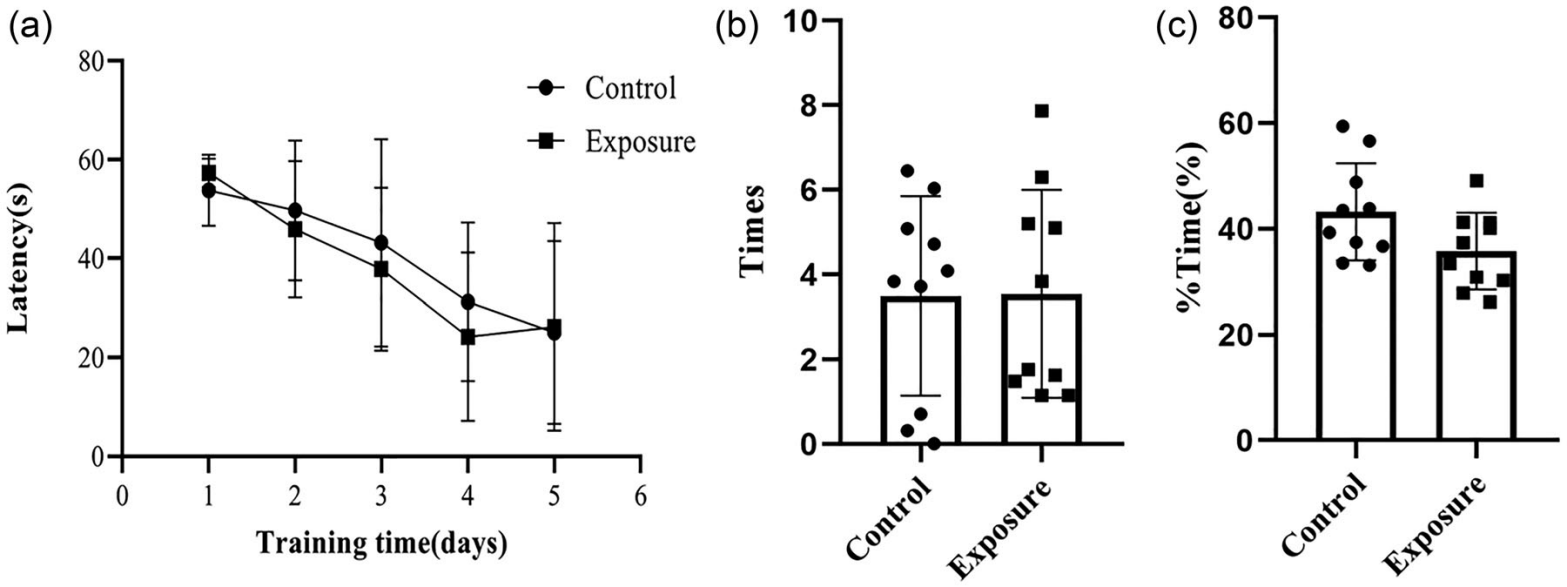
Effect of 2650‐MHz‐EMR exposure on the spatial memory of mice in the Morris water maze (*n* = 10). (A) The escape latency in acquisition phase training; (B) the number of times the mice crossed the original platform in the probe phase; (C) the exploration time in the target quadrant in the probe phase. There was no difference between the control group and the EMR exposure group. All results are expressed as the mean ± SD, and there was no significant difference between the control and exposure groups (*p* > .05).

We used the Morris water maze test to evaluate whether exposure to 2650 MHz‐EMR had an effect on the spatial memory of mice. In acquisition phase training, as the training time was prolonged, the escape latency of both groups of mice decreased (*p* = .627), but there was no statistically significant difference (Figure 9A). After the acquisition phase training, we conducted a probe trial test to further evaluate the changes in the spatial memory function of mice. During the probe phase, we observed the number of times the mice crossed the original platform (*p* = .654) and the exploration time in the target quadrant (*p* = .603), but there were no statistically significant differences between the control and exposure groups (Figure 9B and C). The results of the Morris water maze test suggest that exposure to 2650 MHz‐EMR does not affect the spatial memory function of mice.

### 3.4 Effect of 2650 MHz‐EMR on depression‐like behavior of mice

**FIGURE 10:**
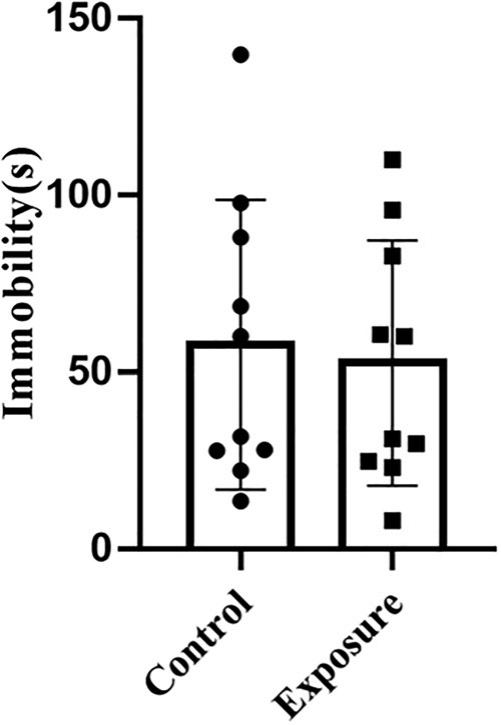
The effect of 2650‐MHz‐EMR exposure on immobility in the tail suspension test (*n* = 10). All results are presented as the mean ± SD, and there was no difference between the control and exposure groups (*p* > .05).

To evaluate whether prolonged exposure to 2650 MHz‐EMR causes not only anxiety‐like behavior in mice but also depression‐like behavior, we used a tail suspension test (Figure 10). There was no statistically significant difference between the two groups of mice in the last 4 min of immobility (*p* = .847), suggesting that exposure to EMR does not cause depression‐like behavior in mice.

### 3.5 Effect of 2650 MHz‐EMR on hippocampal dentate gyrus cells

**FIGURE 11:**
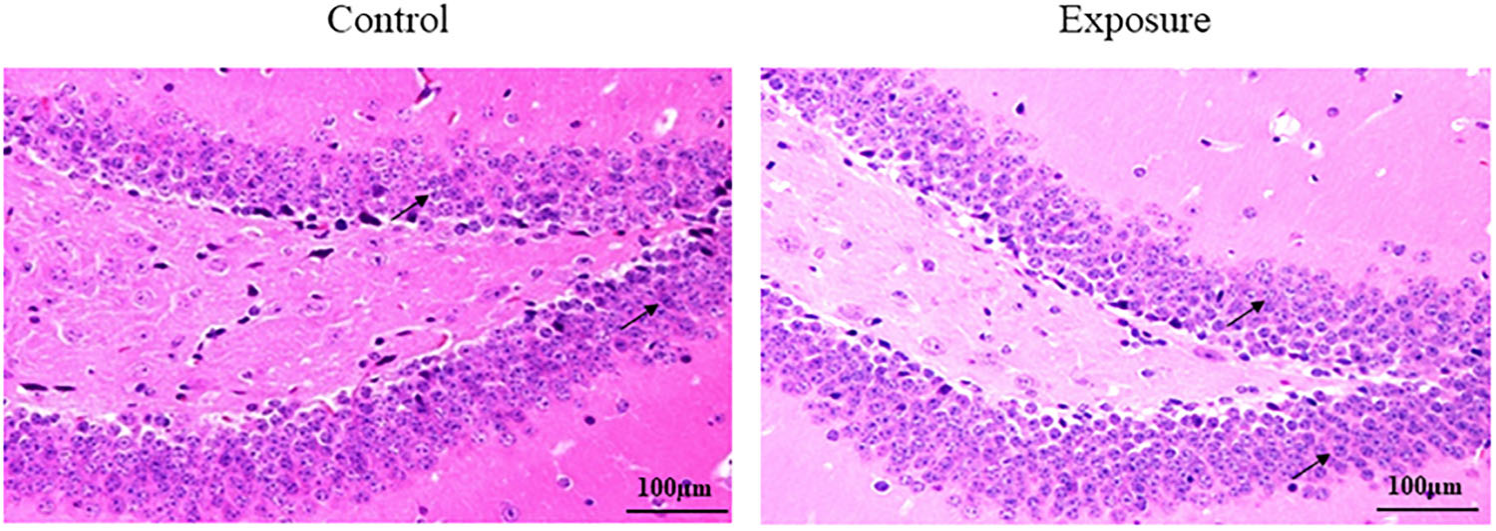
The effect of 2650‐MHz‐EMR exposure on the histopathology of hippocampal tissues. (*n* = 5). There was no difference in the number and morphological changes of hippocampal cells between the control and exposure groups. Black arrows are cells.

We used HE staining to observe whether exposure to 2650 MHz‐EMR has an effect on the morphological changes in hippocampal cells (Figure 11). There was no significant change in the number and morphology of hippocampal cells in the two groups of mice (control group, 407.8 ± 70.4; exposure group, 424.0 ± 53.9), indicating that exposure to EMR had no significant effect on the morphology of hippocampal cells.

### 3.6 Effect of 2650 MHz‐EMR on apoptosis of hippocampal tissues

**FIGURE 12:**
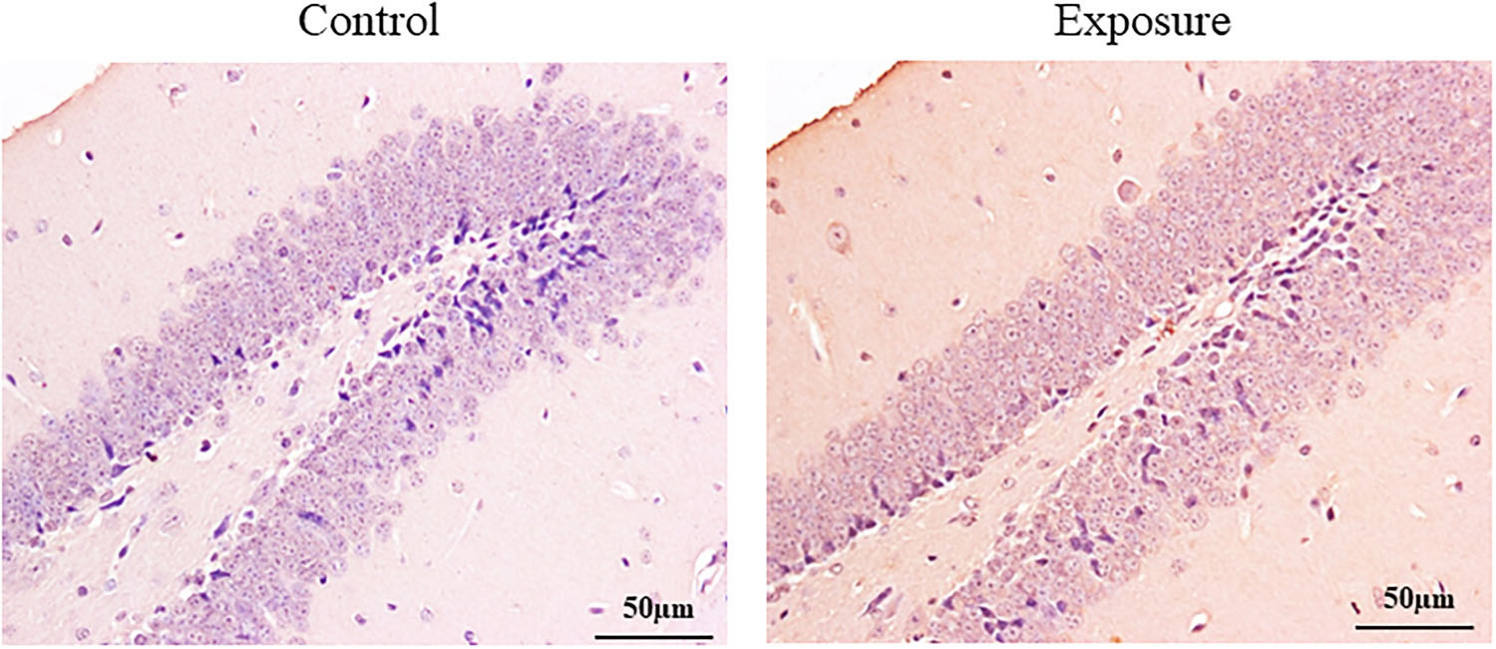
The effect of 2650‐MHz‐EMR exposure on apoptosis of hippocampal tissues. (*n* = 5). The results of apoptosis staining showed that there was no significant difference between the control group and the EMR exposure group. Black arrows are apoptotic cells.

We used TUNEL staining to observe whether exposure to 2650 MHz‐EMR affects apoptosis in the hippocampus (Figure 12). There was no obvious apoptosis of hippocampal cells in either group of mice (control group, 5.00 ± 1.58; exposure group, 6.40 ± 1.14), indicating that exposure to EMR had no significant effect.

### 3.7 Effect of 2650 MHz‐EMR on the expression of BDNF, GR, and GABA in hippocampal tissue

**FIGURE 13:**
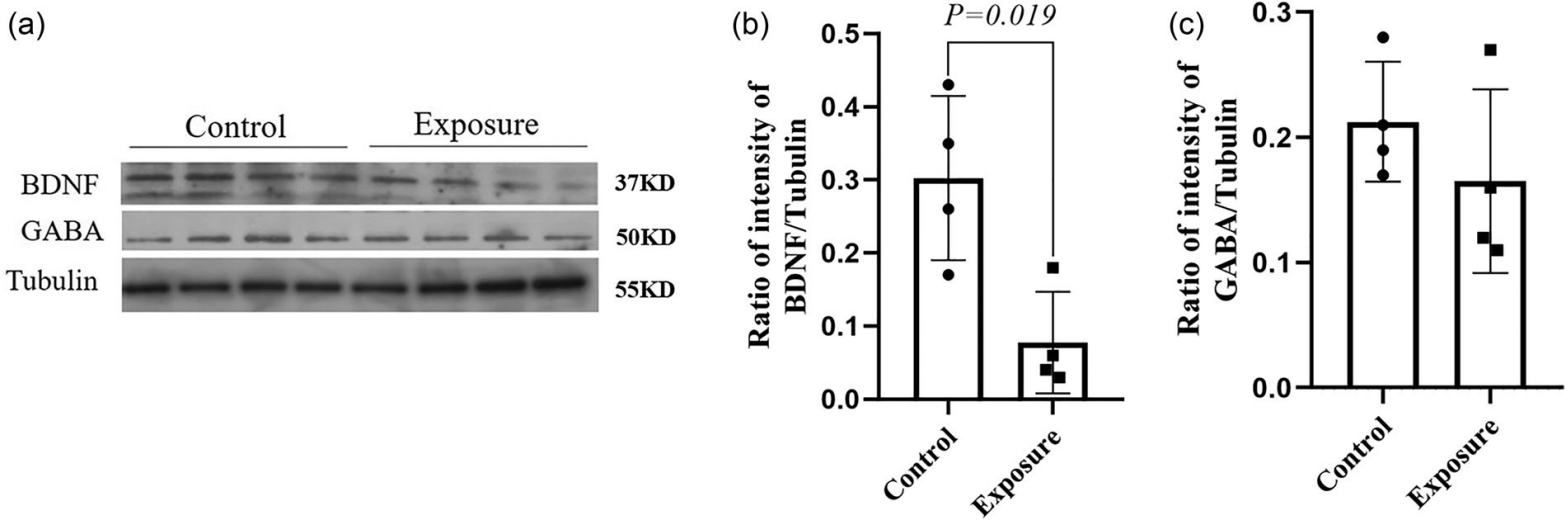
The effect of 2650 MHz‐EMR on hippocampal expression of BDNF and GABA (*n* = 4). (A) Western blot reaction protein bands of BDNF and GABA from hippocampal tissue. Tubulin protein bands were used as internal controls for each gel. (B) BDNF; (C) GABA. The band quantification is expressed as the mean ± SD relative to the relative intensity of tubulin.

**FIGURE 14:**
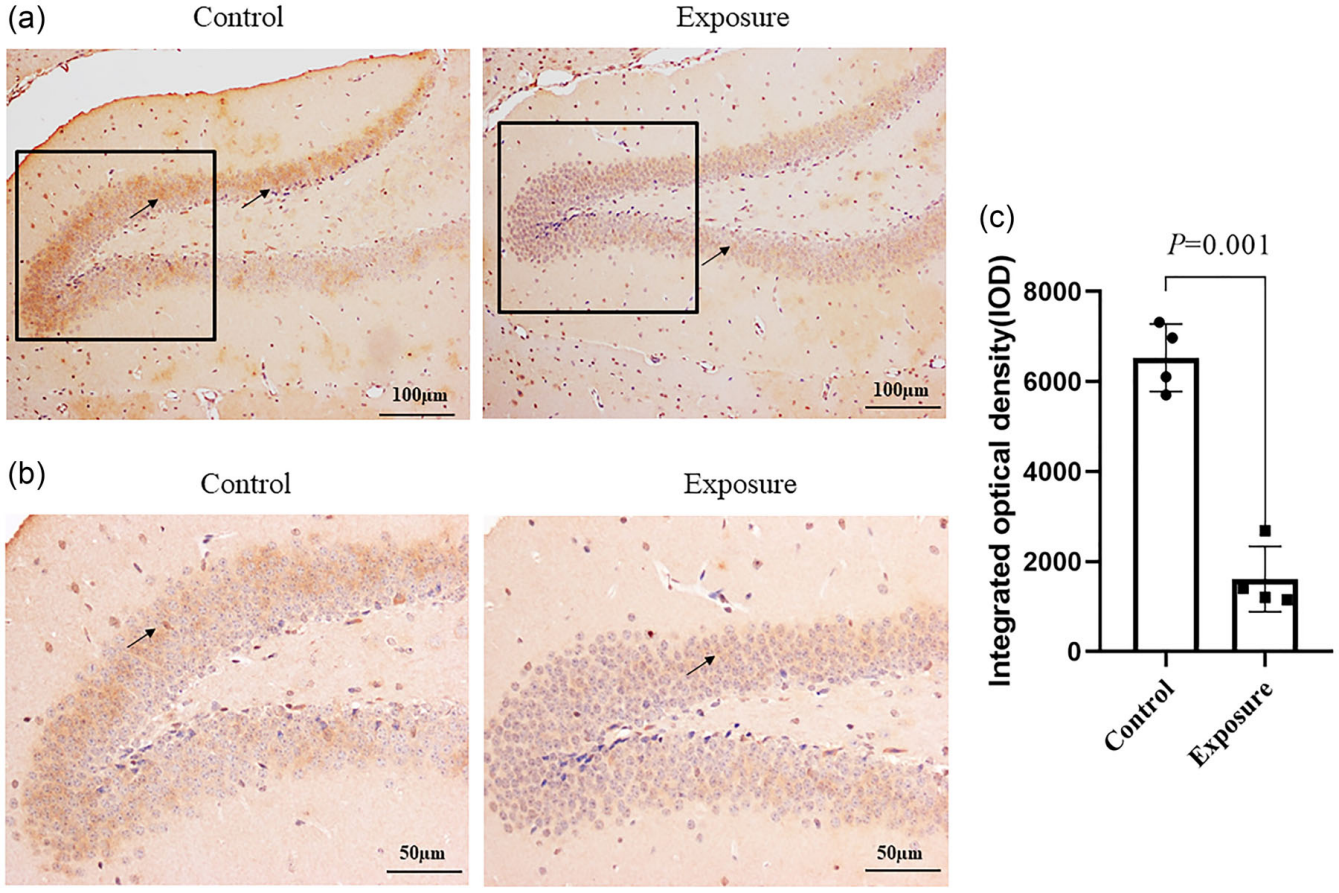
The expression of GR receptor protein in the mouse hippocampus after 2650‐MHz‐EMR exposure (*n* = 4). (A) A 100‐fold picture. (B) A 200‐fold picture in the black frame area of A. Black arrows indicate positive expression. (C) The quantitative analysis of GR expression. All results are expressed as the mean ± SD. Compared with the control group, the expression of GR protein in the EMR exposure group was significantly reduced (*p* < .01).

To further validate the effects of exposure to 2650 MHz‐EMR on the emotional behavior and learning and memory ability of mice, western blotting and immunohistochemistry were used to analyze the expression of related proteins in the brain tissues of mice (Figures 13A–C and 14A–C). The expression of BDNF in the hippocampus of mice exposed to EMR was significantly downregulated by 2650 MHz‐EMR (*p* = .019); however, there was no statistically significant difference in the expression of GABA (*p* = .330). Compared with the control group, the expression of GR protein in the hippocampus of the EMR exposure group was significantly decreased (*p* = .001).

## 4 DISCUSSION

Given that the previous literature has tended to focus on the effects of 3G and 4G mobile phone EMR exposure (Saikhedkar et al., 2014; Wang et al., 2017), we aimed to determine whether 5G mobile phone EMR exposure will have similar effects. In this study, our results suggest that using RC to simulate EMR from mobile phones to expose mice for 28 days may be associated with the anxiety‐like behavior of mice, which is consistent with previous reports (Gupta et al., 2019). The results showed that in the OFT, EMR exposure was significantly associated with a reduction in the total activity distance, central region residence time, and central region activity distance of mice. In EPM, the percentage of the number of times to open the arm and the percentage of time to open the arm in the exposure group were significantly lower than those in the control group. This demonstrates that under the current experimental conditions (4 h per day for 28 days, WBSAR: 2.06 W/kg), EMR exposure was associated with anxiety‐like behavior in mice. Among them, the adjustment of the HPA axis is one of the important mechanisms to maintain the dynamic balance of the internal environment (Kenny et al., 2014). EMR exposure can be regarded as a source of stress, which can activate the HPA axis, increase GR secretion, and further activate a negative feedback regulation system of the HPA axis, thereby helping the body effectively resist external stress. Our results show that the expression of GR protein in the hippocampus of the EMR‐exposed group was significantly reduced. This may be due to the weakening of the negative feedback regulation of the HPA axis after EMR exposure, resulting in continuous hyperactivity of the HPA axis, and provides histology for the weakening of the negative feedback regulation function of the HPA axis. On the other hand, it may also be caused by an uncoupled HPA axis. In addition, CORT is a functional indicator of stress responses in laboratory animals, and CORT levels are susceptible to changes in external pressure, diet, and circadian laws. Compared with the sham operation group, the mice in the experimental group supplemented with low‐dose CORT after bilateral adrenalectomy had no difference in the CORT level under the standard environment, but the experimental group could significantly reduce the CORT level in the enriched environment (Xu et al., 2009). Although it is difficult to objectively quantify the stress situation of mice to obtain accurate CORT levels, in this study, the CORT levels of mice were significantly increased, consistent with previous reports (Gong et al., 2015; Gupta et al., 2019). However, after exposure to EMR for up to 28 days, we did not observe depression‐like behavior in mice, suggesting that EMR exposure may not be related to depression‐like behavior under the conditions of this study.

BDNF is a major member of the neurotrophin family. It is widely distributed in the brain, especially in the hippocampus, and its expression in the hippocampus is closely related to anxiety‐like symptoms (Govindarajan et al., 2006; Lindsay et al., 1994). BDNF plays an important role in promoting the growth and differentiation of immature metagenesis, the survival and maintenance of mature neurons, and improving neuroplasticity and antidepressant treatment during the occurrence of depression (Zhang et al., 2017). Therefore, the change in BDNF expression level is closely related to the occurrence of anxiety disorder. Compared with nonalcohol‐preferring rats, alcohol‐preferring rats showed significant anxiety behavior, and the activity of BDNF and its downstream target molecules regulated the expression of mRNA and protein levels of cytoskeletal proteins (Moonat et al., 2011). In addition, the observation of 324 people found that the low serum content of BDNF was significantly correlated with the anxiety disorder reflected by the temperament characteristics survey (*p* = .009), so that the reduction of serum BDNF content may be a biochemical sign of anxiety (Minelli et al., 2011). An increase in BDNF was associated with improved anxiety. According to previous reports, the rat anxiety model is obtained after prolonged low‐current shock or forced swimming, and then the anxiety level is reduced by injecting BDNF into the spinal cord of anxious rats (Siuciak et al., 1997). Similarly, our results indicate that EMR may be associated with anxiety‐like behavior in mice, resulting in a significant downregulation of hippocampal BDNF expression, which is consistent with most reports (Antipova et al., 2009; Li et al., 2010). Thus, we may infer that the degree of anxiety‐like behaviors in mice is closely related to the decreased expression of BDNF, although further experiments are needed to explore the more detailed mechanism of action.

Learning and memory is an advanced function of the brain, and its ability is regulated by many factors. At present, many studies have focused on synaptic plasticity and neurotransmitters. Long‐term potentiation (LTP) of synaptic plasticity is considered to be the main neural basis for regulating learning and memory (Solomonia & Mccabe, 2015). There are excitatory and inhibitory transmitters in the brain, among which glutamic acid (Glu) is the most important excitatory transmitter, and the inhibitory transmitter is mainly alpha‐aminobutyric acid (GABA) (Wu & Sun, 2015). When learning signals are transmitted into the brain, Glu is induced to release, a small amount of Glu promotes learning and memory, and an excessive amount of Glu can cause neurotoxicity (Auger & Floresco, 2017); however, GABA also participates in the regulation of LTP, which has a certain inhibitory effect on learning and memory. GABA participates in the process of learning and memory mainly by acting on the postsynaptic membrane to cause the influx of chloride ions, inhibit the production of neuronal excitation, and thus regulate various cognitive functions such as learning and memory (Paine et al., 2015). Studies have shown that in the water maze model, the spatial learning ability of transgenic mice lacking the 5‐subunit of GABA A is significantly improved (Collinson et al., 2002). When the expression level of GABA is increased, it negatively inhibits the spatial learning ability of mice. In this study, we used the MWM test to investigate the effects of EMR on spatial memory in mice. The results showed that there was no significant difference between the EMR exposure group and the control group in the incubation period of successful escape, suggesting that under such conditions (SAR: 2.06 W/kg, 28 days, 4 h per day), there was no negative effect on the spatial memory ability of mice. However, it is worth mentioning here that in EMR exposure of mice, the time to find a hidden platform underwater is relatively short. Likewise, there was no difference in the expression of hippocampal GABA between the two groups, which was consistent with that reported in the literature. In addition, it has been reported that exposure to EMR induces apoptosis, degeneration of hippocampal neurons, and a significant decrease in the number of neurites (Del Vecchio, Giuliani et al. 2009; Ertilav et al., 2018; Hussein et al., 2016). However, we used HE staining and TUNEL staining to observe the changes in the number and morphology of hippocampal cells and found no difference between the EMR exposure group and the control group. This may be related to the selected radiation time and power in this study. To further clarify the damaging effect of EMR on learning and memory in mice, it is necessary to increase the radiation power or extend the radiation time.

## 5 CONCLUSION

In conclusion, our research suggests that exposure to 2650 MHz‐EMR (WBSAR: 2.06 W/kg, 28 days, 4 h per day) had no significant effect on the spatial memory ability of mice (in comparison to little effect). It may be associated with anxiety‐like behavior in mice but not related to depression‐like behavior in mice. These findings will help to provide a reference for the protection of people receiving high EMR exposure. Although in this study, we found that anxiety‐like behaviors in mice may be associated with EMR, more investigations are still needed to confirm the results.

## AUTHOR CONTRIBUTIONS

**Dawen Gao**: Data curation; **Yan Gao**: Formal analysis; **Guofu Dong**: Investigation; **Wenjing Gong**: Validation; **Rongqi Zheng**: Writing—original draft; **Xianxie Zhang**: Data analysis and chart making and others; **Zhihui Li and Chenggang Zhang**: Writing—review & editing

## CONFLICT OF INTEREST STATEMENT

The authors declare no conflict of interest.

### FUNDING

Chenggang Zhang: 2012CB518200; Chenggang Zhang: 81371232, 81573251; Chenggang Zhang: 2012ZX09102301‐016, 2014ZX09J14107‐05B; Xianxie Zhang: 82004054.

### PEER REVIEW

The peer review history for this article is available at https://publons.com/publon/10.1002/brb3.3004

## Data Availability

The data used to support the findings of this study are available from the corresponding author upon request.
